# Arterial waveform parameters in a large, population-based sample of adults: relationships with ethnicity and lifestyle factors

**DOI:** 10.1038/jhh.2016.78

**Published:** 2016-12-22

**Authors:** J D Sluyter, A D Hughes, S A McG Thom, A Lowe, C A Camargo Jr, B Hametner, S Wassertheurer, K H Parker, R K R Scragg

**Affiliations:** 1 School of Population Health, University of Auckland, Auckland, New Zealand; 2 Institute of Cardiovascular Sciences, University College London, London, UK; 3 International Centre for Circulatory Health, Imperial College London, London, UK; 4 Institute for Biomedical Technologies, Auckland University of Technology, Auckland, New Zealand; 5 Department of Emergency Medicine, Massachusetts General Hospital, Harvard Medical School, Boston, USA; 6 Health & Environment Department, AIT Austrian Institute of Technology, Vienna, Austria; 7 Department of Bioengineering, Imperial College London, London, UK

## Abstract

Little is known about how aortic waveform parameters vary with ethnicity and lifestyle factors. We investigated these issues in a large, population-based sample. We carried out a cross-sectional analysis of 4798 men and women, aged 50–84 years from Auckland, New Zealand. Participants were 3961 European, 321 Pacific, 266 Maori and 250 South Asian people. We assessed modifiable lifestyle factors via questionnaires, and measured body mass index (BMI) and brachial blood pressure (BP). Suprasystolic oscillometry was used to derive aortic pressure, from which several haemodynamic parameters were calculated. Heavy alcohol consumption and BMI were positively related to most waveform parameters. Current smokers had higher levels of aortic augmentation index than non-smokers (difference=3.7%, *P*<0.0001). Aortic waveform parameters, controlling for demographics, antihypertensives, diabetes and cardiovascular disease (CVD), were higher in non-Europeans than in Europeans. Further adjustment for brachial BP or lifestyle factors (particularly BMI) reduced many differences but several remained. Despite even further adjustment for mean arterial pressure, pulse rate, height and total:high-density lipoprotein cholesterol, compared with Europeans, South Asians had higher levels of all measured aortic waveform parameters (for example, for backward pressure amplitude: *β*=1.5 mm Hg; *P*<0.0001), whereas Pacific people had 9% higher log_e_ (excess pressure integral) (*P*<0.0001). In conclusion, aortic waveform parameters varied with ethnicity in line with the greater prevalence of CVD among non-white populations. Generally, this was true even after accounting for brachial BP, suggesting that waveform parameters may have increased usefulness in capturing ethnic variations in cardiovascular risk. Heavy alcohol consumption, smoking and especially BMI may partially contribute to elevated levels of these parameters.

## Introduction

Cardiovascular disease (CVD) prevalence is increased in Polynesian and South Asian people relative to white/European populations.^1, 2, 3, 4^ This may be mediated by ethnic differences in blood pressure (BP) and other arterially related parameters and, as the latter may be clinically important,^5, 6, 7^ interventions directed at improving arterial function might reduce these CVD disparities. In addition, an understanding of ethnic variations in arterial function may help to identify the population groups to be targeted in public health interventions.

New methods^8^ have expanded the scope of measurable variables from the aortic pressure waveform such as aortic BP, augmentation index (Alx), backward pressure (Pb) amplitude and excess pressure integral (EPI).^5, 6, 7^ These parameters are important because they have been shown to predict CVD incidence independently of and more strongly than brachial BP.^5, 6, 7^ A few studies have examined differences in aortic waveform parameters between South Asian and European people,^9, 10, 11^ although these studies did not assess parameters derived from wave separation^6^ or reservoir wave^7^ analyses. Another drawback is that some of these studies investigated selected populations that either excluded people with high BP^10^ or included only those with BP-related morbidity (such as patients with heart failure^11^). This restriction may result in poorly representative samples and the inability to fully explore the relationship between ethnicity and arterial function. In addition, we are not aware of any studies that have evaluated differences between Polynesian and European people.

Identifying determinants of arterial waveform measures helps to define suitable strategies for improving arterial function. Three possible determinants are alcohol consumption, smoking and body mass index (BMI). A meta-analysis showed that alcohol reduction decreased brachial BP among heavy drinkers,^12^ cigarette smoking has been shown to increase brachial BP,^13^ whereas smoking cessation has been demonstrated to reduce brachial BP,^14^ and a meta-analysis showed that weight loss reduced brachial BP.^15^ Although these intervention studies demonstrate that reducing these three factors decreases brachial BP, it is not known whether they are associated with various aortic waveform parameters.

The first objective of this study was to explore ethnic differences in arterial waveform parameters between Europeans and people from Polynesian and South Asian groups. We aimed to include a wide range of arterial function measures, which may reveal new ethnic variations. Our second objective was to examine associations between these parameters and alcohol consumption, smoking and BMI.

## Materials and methods

### Participants

The present study is a baseline (cross-sectional) analysis of the ViDA (Vitamin D Assessment) study, a randomised controlled trial of the health effects of vitamin D supplementation in 5110 people. Participants were recruited from patient registers of family doctors in Auckland. Inclusion criteria were men and women aged 50–84 years and resident in Auckland at recruitment. Participants were excluded if they had been diagnosed with a terminal illness and/or in hospice care, intended to leave New Zealand during the follow-up period, took vitamin D supplements (including cod liver oil) of >600 IU per day, had a history of specific medical conditions (renal stones, hypercalcaemia or conditions that can cause hypercalcaemia) and/or had a baseline serum calcium >2.50 mmol l^−1^. All baseline data were collected between 2011 and 2012. Ethics approval was provided by the Ministry of Health Multi-region Ethics committee. Written, informed consent was obtained from each participant. Full details of the study design have been published elsewhere.^16^

### Measurements other than BP parameters

Demographic and lifestyle information, along with both history of diabetes and CVD, were collected via questionnaires administered by trained interviewers using a standardised protocol. Ethnicity was defined by self-identification. Participants were initially categorised into four main ethnic groups: Pacific, Maori (both Polynesian groups), South Asian and European/Other. Subsequently, we excluded 'Other' (non-European) people from the 'European/Other' group as we aimed to have Europeans only in our ethnic comparisons. Brachial BP has been found to vary between individual Pacific groups.^3^ Therefore, in the current study, the Pacific group was further broken down into specific categories of adequate sample sizes: Samoan, Tongan and Other Pacific (those from other Pacific groups).

Alcohol consumption was assessed with the question, 'How often do you have six or more drinks on one occasion?' Responses were categorised into three groups: 'Never', '⩽1 per month' and 'weekly, daily or almost daily'. Smoking was assessed by asking participants whether they had previously smoked cigarettes, and whether they described themselves as ex-smokers or current smokers. Responses were classified as 'non-smoker', 'ex-smoker' or 'current smoker.' Participants were also asked the number of years since quitting (for ex-smokers) and the number of cigarettes smoked per day.

To adjust for the effect of antihypertensive medications on the waveform parameters, details of all prescriptions dispensed for participants before and after their interview dates were collected from the Ministry of Health. To determine that measured BP parameters have been influenced by prescribed drugs, these medicines must have been taken just prior to the interview. Therefore, antihypertensive use was defined as the prescription of an antihypertensive drug with days of supply that encompassed the interview date.

Height was measured with a stadiometer to the nearest 0.1 cm, and weight with digital scales to the nearest 0.1 kg. These measurements were made without shoes and in light clothing. BMI was calculated as body weight (kg) per height (m).^2^ A blood sample was taken, and collected aliquots were stored at −80 °C (−112 °F) and later measured for both serum total cholesterol and HDL (high-density lipoprotein) cholesterol on a Siemens Advia 2400 analyser (Siemens Healthcare Diagnostics, Erlangen, Germany).

### Arterial waveform measures

After 15 min rest while sitting, BP (±1 mm Hg) was measured three times with an Omron T9P oscillometric device (Omron Healthcare, Kyoto, Japan) placed above the cubital fossa of the left arm and the mean of the two closest measurements were used for analyses. Suprasystolic oscillometry was carried out using a BP+ device (Uscom, Sydney, Australia) (formerly known as a *R6.5* cardiovascular monitor; Pulsecor, Auckland, New Zealand), with an appropriately sized cuff positioned over the left upper arm. The BP+ device has been shown to: (1) yield central systolic BPs that are highly correlated with those assessed by catheter measurement at the ascending aorta or aortic arch^17^ and, (2) measure central systolic BP with good intratest and intertest reliability (intraclass correlation coefficients=0.975 and 0.895, respectively).^18^ To improve the quality of the waveforms used in analyses, we decided *a priori* to exclude readings with a signal-to-noise ratio of <6 dB.

AIx, an index of arterial stiffness and wave reflection,^19^ was calculated from the aortic pressure waveform using custom-written Matlab software (Mathworks, Natick, MA, USA). A meta-analysis has shown AIx to predict CV events.^5^

Wave separation analysis was used to separate the aortic pressure waveform into forward and backward components.^20^ The amplitude of backward pressure (Pb) was then calculated. Pb determined from this technique has previously been shown to be similar to values obtained using aortic flow waveforms measured by Doppler ultrasound.^21^ Further, independently of brachial BP, Pb has been shown to predict mortality^22^ and CV events.^6^

Aortic pressure was separated into reservoir and excess components using custom-written Matlab software (Mathworks). Reservoir pressure was calculated from pressure measurements, as described elsewhere.^7^ Peak reservoir pressure was calculated as the amplitude of the reservoir pressure waveform, which has been found to associate positively with the risk of cardiovascular events independently of brachial BP.^20^ Excess pressure was calculated as measured pressure minus reservoir pressure.^23^ The integral of the excess pressure waveforms (area under these waveforms) over the cardiac cycle was used to calculate EPI. EPI measures pressure that is associated with excess ventricular work and has been shown to predict CV events independently of brachial SBP.^7^

Aortic pulse wave velocity (PWV) was calculated from validated algorithms and derived PWV values have been shown to predict CV events independently of brachial BP.^24, 25^ PWV is a known predictor of CV events, as demonstrated in a meta-analysis.^26^

### Statistical analysis

Equality of variance across groups was checked with Levene’s test. Inter-ethnic differences in participant characteristics were assessed with analysis of variance (for continuous variables) and *χ*^2^-tests (for categorical variables). Because of the positively skewed distribution of EPI, this was converted to log_e_ for analyses. Associations between independent variables and arterial waveform parameters were examined by multivariate linear regression, adjusting for covariates (listed in footnotes of tables) that are determinants of BP variables. In separate models and in accordance with research similar to ours,^27^ correction was made for pulse rate as this influences the timing of reflected pressure waves and is a known contributor to the shape of the aortic pressure waveform.^28^ The significance of main effects was examined with the *F*-test. Potential interactions between sex and independent variables of these waveform relationships were examined but as these were not statistically significant, we did not report sex-specific results. All analyses were performed using SAS version 9.3 (SAS Institute, Cary, NC, USA) and statistical significance was set at *P*<0.05 (two-sided).

## Results

### Participant characteristics

Table 1 shows the characteristics of the participants across the ethnic groups. A total of 4798 people were included in the analyses, although not all individuals answered questions so that the total sample sizes for each of the categorical variables varied slightly (maximum difference was *n*=41 or <1% of the total sample size, indicating a minimal loss of data). The participants were 3961 Europeans, 321 Pacific people, 266 Maori and 250 South Asians. The composition of the Pacific group was as follows: 105 (33%) Samoan, 82 (26%) Tongan and 134 (42%) Other Pacific. The Other Pacific group predominantly comprised Cook Island Maori people (57%); the remainder being Niuean (25%), Indigenous Fijian (12%), Tahitian (1%) and Other (5%).

As shown in Table 1, heavy alcohol consumption varied with ethnicity (*P*<0.0001). Smoking prevalence (combined proportion of ex-smokers and current smokers) was higher in Maori and lower in South Asians with respect to Europeans (*P*<0.0001 for relationship between smoking and ethnicity). Supplementary analyses (not tabulated) revealed that, on average (median value), ex-smokers quit smoking 28 years ago (interquartile range: 22 years), and the median number of cigarettes smoked per day among current smokers was approximately just under 10 (58% smoked ⩽10 per day). Compared with that of Europeans, BMI was higher in both Maori and Pacific but lower in South Asians (*P*<0.0001 for variation in BMI with ethnicity; Table 1).

### Ethnic differences in arterial waveform parameters

Ethnic differences in arterial waveform parameters between European and non-European groups are shown in Table 2. Three sets of comparisons were made: those unadjusted for brachial BP, adjusted for brachial SBP and adjusted for brachial DBP. For the comparisons unadjusted for brachial BP, all parameters varied with ethnicity (all *P*⩽0.042). Compared with Europeans, Maori had higher levels of brachial SBP, brachial DBP, aortic SBP, AIx, Pb, peak reservoir pressure and log_e_(EPI). Pacific people had higher levels of brachial SBP, brachial DBP, aortic SBP, Pb, peak reservoir pressure, log_e_(EPI) and PWV with respect to Europeans. Relative to Europeans, South Asians had higher levels of AIx, Pb and log_e_(EPI).

For the comparisons adjusted for brachial SBP (Table 2), all parameters varied with ethnicity (all *P*⩽0.004), except for aortic SBP (*P*=0.32) and peak reservoir pressure (*P*=0.76). At a given brachial SBP, compared with Europeans, South Asians had higher levels of AIx, Pb and log_e_(EPI). Compared with Europeans, at the same brachial SBP, Maori had lower PWV, whereas Pacific people had higher log_e_(EPI) but lower AIx and PWV.

Similarly, at a fixed brachial DBP, all waveform parameters varied with ethnicity (all *P*⩽0.034; Table 2), except for PWV (*P*=0.50). For the same brachial DBP, South Asians had higher levels of all waveform parameters relative to Europeans, except for PWV. With respect to Europeans, Pacific people had higher levels of aortic SBP and log_e_(EPI), whereas Maori had higher levels of log_e_(EPI).

We additionally examined differences in arterial waveform parameters within Pacific groups (having Other Pacific as the reference group; results not tabulated), adjusted for age, sex, antihypertensive use, diabetes and CVD (plus pulse rate for AIx). AIx varied across the Pacific groups (*P*=0.013), with Other Pacific people having higher AIx than both Samoans (*β*=3.6%, *P*=0.011) and Tongans (*β*=3.7%, *P*=0.016). There were no other differences among the Pacific groups (all *P*>0.05).

### Relationships between lifestyle factors and arterial waveform parameters

Adjusted associations between modifiable factors and arterial waveform parameters are illustrated in Table 3. Frequency of heavy alcohol consumption had positive, dose-dependent relationships with brachial SBP, aortic SBP, peak reservoir pressure and PWV (all *P*⩽0.005). In each case, the beta-coefficients for people who consumed the most were more than double that of those whose consumption was ⩽1 per month. AIx varied with smoking (*P*<0.0001), with smokers having higher levels of this variable than non-smokers. Specifically, AIx was higher in ex-smokers than in non-smokers (*β*=0.6%, *P*=0.4) but notably even more so in current smokers (*β*=3.7%, *P*<0.0001). BMI had positive relationships with all parameters except AIx and Pb (all *P*<0.0001).

Pulse rate was positively associated with heavy alcohol consumption (*P*=0.004), smoking (*P*<0.0001) and BMI (*P*<0.0001) (adjusted for age, sex, ethnicity, antihypertensive use, diabetes and CVD). When the models for Table 3 were also adjusted for pulse rate, the relationships were largely unchanged. However, new associations were observed in a few cases. That is, heavy alcohol consumption now had positive relationships with Pb (*P*=0.015) and log_e_(EPI) (*P*=0.007), whereas smokers (particularly current ones) had higher log_e_(EPI) than non-smokers (*P*=0.0002).

### Impact of lifestyle and physiological variables on ethnic differences

Next we examined the individual contributions of BMI, heavy alcohol consumption and smoking to the ethnic differences (controlling for sex, age, antihypertensive use, diabetes and CVD, plus pulse rate for AIx; results not illustrated). Maori and Pacific people had higher levels of arterial waveform parameters than Europeans but, in most cases, these differences were markedly attenuated (by between 11% and 72%) when BMI was controlled for. Adjustment for heavy alcohol consumption had a predominantly minor impact on these differences, changing these by between 2% and 15%. Controlling for smoking resulted in primarily small changes (by <1–12%) to the Pacific, Maori and South Asian regression coefficients.

**Figure 1:**
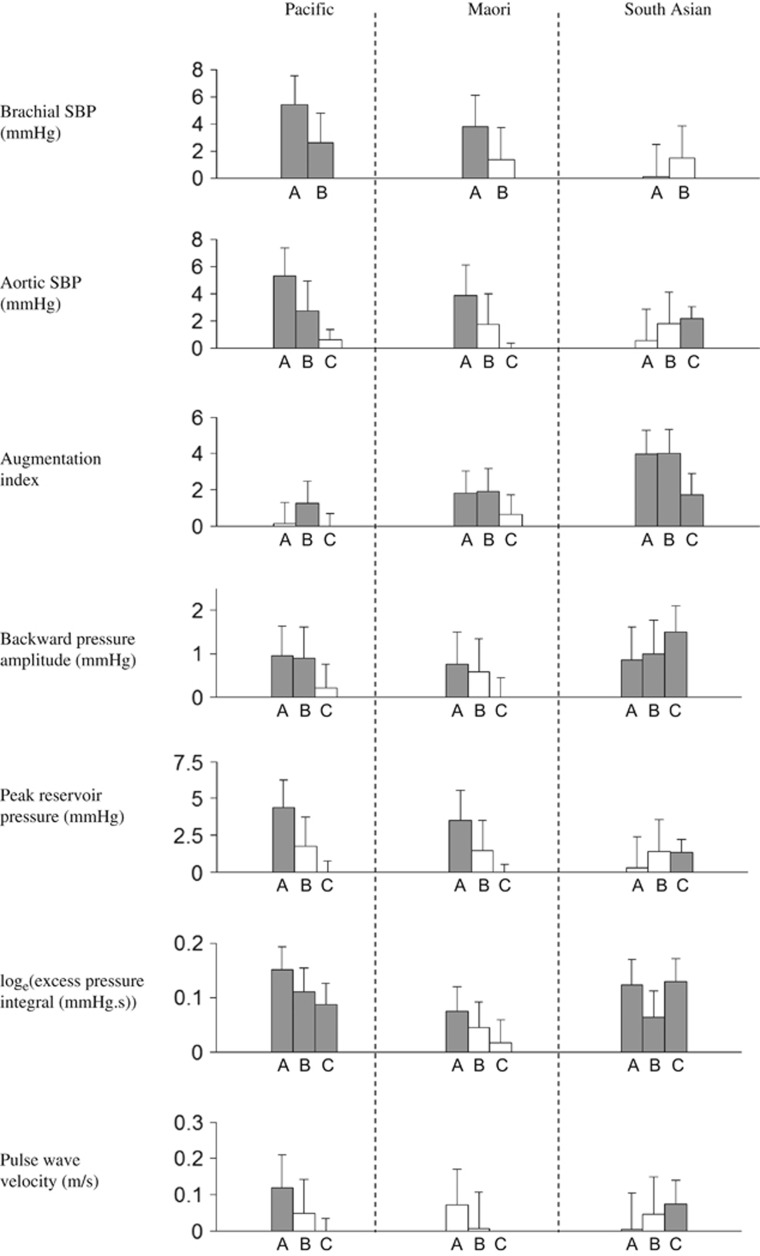
Ethnic differences, compared with Europeans, in arterial waveform parameters. Bar A covariates are age, sex, antihypertensive use, diabetes and cardiovascular disease, plus pulse rate for augmentation index. Bar B covariates are bar A covariates plus heavy alcohol consumption, smoking and BMI. Bar C covariates are bar B covariates plus mean arterial pressure, pulse rate, height and total:HDL cholesterol ratio. Grey and white bars represent significant and non-significant differences, respectively. Error bars represent the upper values of the 95% confidence intervals.

Following this, we evaluated the cumulative contributions of several variables to the ethnic differences (Figure 1). As illustrated in this figure, after controlling for age, sex, antihypertensive use, diabetes and CVD, non-European people had higher levels of arterial waveform parameters than Europeans in most cases. Additional correction for all three lifestyle factors (heavy alcohol consumption, smoking and BMI) notably reduced Maori and Pacific differences with European. But several ethnic differences nevertheless persisted. We adjusted even further for the following physiological variables: mean arterial pressure, pulse rate, height and total:HDL cholesterol ratio. This reduced Maori and Pacific differences but Pacific people still had higher log_e_(EPI) (*β*=0.09, indicating that EPI was e^0.09^ or 1.09 times higher; *P*<0.0001) than Europeans. In contrast, after this adjustment, South Asians had higher levels of aortic SBP (*β*=2.2 mm Hg; *P*<0.0001), AIx (*β*=1.7% *P*=0.0028), Pb (*β*=1.5 mm Hg; *P*<0.0001), peak reservoir pressure (*β*=1.3 mm Hg; *P*=0.0053), log_e_(EPI) (*β*=0.13, indicating that EPI was e^0.13^ or 1.14 times higher; *P*<0.0001) and PWV (*β*=0.07; *P*=0.028) with respect to Europeans.

## Discussion

Our results show that New Zealanders of non-European ethnicity have higher levels of arterial waveform parameters than those of European ethnicity. For the same brachial SBP or DBP, these parameters varied with ethnicity, with South Asians particularly having higher levels of these measures relative to Europeans. Heavy alcohol consumption and BMI were positively and directly related to nearly all waveform parameters, whereas smoking was positively and directly associated with most of these. These lifestyle factors together made large contributions to a number of ethnic variations in these waveform parameters. But several ethnic differences remained and, despite further accounting for mean arterial pressure, pulse rate, height and total:HDL cholesterol ratio, South Asians nonetheless had higher levels of all aortic waveform parameters relative to Europeans.

The higher levels of brachial SBP we observed among Pacific people relative to Europeans concurs with findings in previous studies.^29, 30^ We extend this prior research by showing that these ethnic groups additionally differ in aortic waveform parameters. Other new ethnic variations were the higher AIx, Pb and EPI we observed among South Asians with respect to Europeans. In line with this, a relatively small study reported South Asians to have higher stiffness index than Europeans.^10^

Another original finding was that, within the Pacific group, there were very few differences in aortic waveform parameters. In accordance with our results, Samoan and Tongan adults have been found to have similar brachial SBP.^3^ Despite this consistency, we cannot rule out the possibility of intra-ethnic differences after consideration of limitations with regard to sample size and representativeness (discussed below).

Given that aortic waveform parameters varied with ethnicity and that they predict cardiovascular events,^5, 6, 7^ they may mediate disparities in CVD prevalence that exist between the ethnic groups studied in this paper.^1, 2, 3, 4^ Therefore, interventions directed at improving arterial function in these populations might reduce these CVD disparities. Further, several of the waveform differences were notable in size. For example, log_e_(EPI) was 0.13 higher in Pacific people than the reference group, indicating that EPI was e^0.13^ or 1.14 times higher (a 14% difference) than among Europeans. Although these results show that the ethnic variations are potentially of clinical significance, longitudinal analyses are required to establish the contribution of these waveform variations to ethnic differences in CVD incidence.

Previous studies have not examined whether, at a fixed brachial SBP or DBP, the ethnic groups we studied have different or similar levels of aortic waveform parameters. Such comparisons are of significance because brachial BP is a widely used measure of arterial function but various central BP parameters may be more important predictors of cardiovascular morbidity and/or mortality.^5, 6, 7^ Thus, we investigated how these parameters vary with ethnicity at a given brachial SBP or DBP. Our finding that they varied with ethnicity suggests that, across ethnic groups, it may be difficult to compare arterial function and its associated cardiovascular risk using brachial BP alone.

Further, these results have implications for defining hypertension or the risks associated with high BP. For example, at a given brachial SBP, South Asians had higher parameter levels than Europeans (Table 2). Therefore, for the same parameter level, they would have *lower* brachial SBP. This indicates that the parameter level at a hypertension threshold of 140 mm Hg of brachial SBP in Europeans, for instance, would correspond to a lower brachial SBP in South Asians. Therefore, to more accurately reflect these parameters (but not necessarily risk of CVD), brachial SBP and DBP cut-off points for defining hypertension may need to be set at different levels for the non-European groups we studied.

Our finding that heavy alcohol consumption and BMI were positively and directly associated with brachial SBP concurs with previous intervention studies.^12, 13, 14, 15^ We extend this prior research by showing that relationships additionally exist for several non-brachial measures (Table 3). The positive association between smoking and EPI (only after adjusting for pulse rate) is another new finding and the higher AIx observed among current smokers is consistent with a small intervention study in young adults.^13^ Compared with non-smokers, current smokers had 3.7 or 12% higher AIx (Table 3); this percentage increment is relatively large and potentially of clinical importance. Altogether, these results support directing interventions at reducing heavy alcohol consumption, smoking and BMI as means to reduce levels of aortic waveform parameters.

Another original finding was the contribution of modifiable factors to ethnic differences in these parameters. While heavy alcohol consumption and smoking had modest contributions, BMI accounted for a large part of the Pacific and Maori differences. This reflects the fact that BMI was strongly associated with waveform parameters (Table 3) and was notably higher in both Pacific and Maori people relative to Europeans (Table 1). Thus, interventions directed at reducing BMI in Polynesian people could be useful for reducing ethnic disparities in the waveform parameters.

Some of the sample sizes for the ethnic minority groups are comparatively small, but they are sufficient to detect (with 90% power and alpha=0.05) differences of ~0.2 s.d.s of waveform parameters, which should be adequate to identify clinically meaningful differences.^5, 6, 7, 20, 22, 26^ Nonetheless, our study would be enhanced by having larger sample sizes of non-European groups. In particular, for the comparisons within Pacific groups, larger sample sizes may be required to detect possible differences. Although our statistical models accounted for known confounders, they are nevertheless subject to residual confounding from unobserved factors. However, as demonstrated above, our results are consistent with those of past research, including intervention studies. Although the current study was population-based, the non-participation of people decreases the external validity of our findings. However, this does not necessarily lead to selection bias and, if it did, the effect may have been reduced by adjustment for the covariates in our models if they were related to non-participation.^31^ Further, with selection bias being more of a problem when non-participation is related to both exposure/independent and outcome variables,^31^ we believe that any bias from the inclusion/exclusion criteria of our study would not be a major issue since it is unlikely that they are strongly related to both the independent and dependent variables in our statistical models. Finally, the study sample was restricted to adult patients of Auckland family doctors and thus our ability to extrapolate findings more widely is limited.

In summary, we found new differences in arterial function between European and non-European people, including measures of wave reflection and excess cardiac work. Several of these, particularly South Asian differences, remained unexplained as they were independent of numerous BP-related factors including demographics, lifestyle and physiological variables. As these varied in line with recognised differences in CVD prevalence between these populations and several were notable in size, the observed variations are potentially important mediators of ethnic CVD disparities. Across ethnic groups, at the same brachial BP, aortic waveform parameters vary with ethnicity, suggesting that the former has limited usefulness in capturing ethnic variations of these parameters. In other words, brachial BP cannot act as a surrogate for these parameters and ethnic differences in the latter will be better monitored by quantifying these parameters (using monitors such as the BP+device used in the current study). Finally, heavy alcohol consumption, smoking and BMI were positively and directly associated with these parameters, supporting interventions aimed at reducing these three risk factors as strategies to improve arterial function. Reducing BMI may be a particularly important strategy as this modifiable factor made a large contribution to ethnic differences in the waveform parameters.


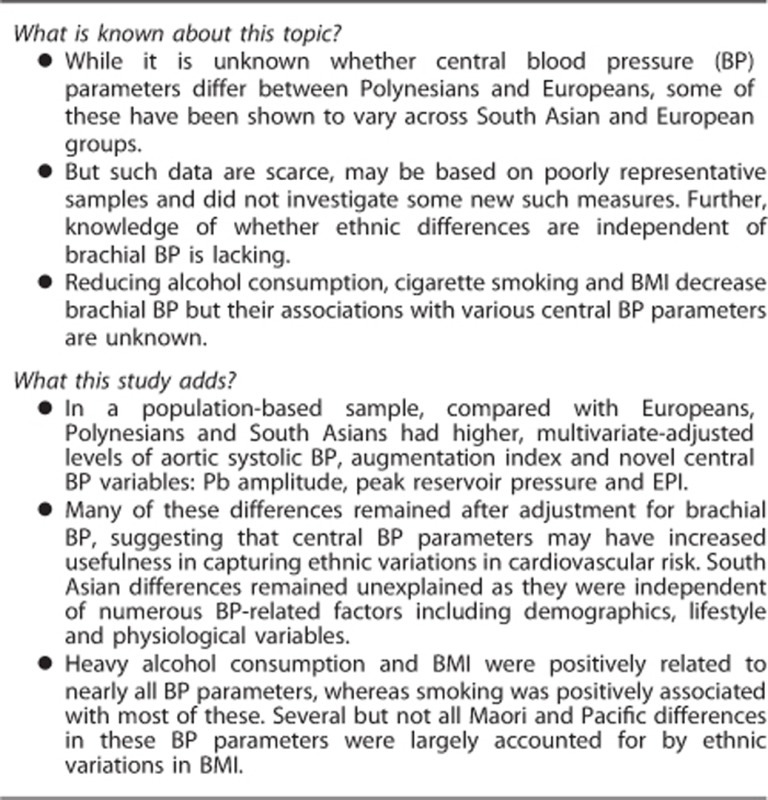


## Figures and Tables

**Table 1:** Characteristics of participants across ethnic groups^a^

*Variable*	*Group*	*European*	*Pacific*	*Maori*	*South Asian*	P*-value*
*n*		3961	321	266	250	
						
Age (years)		67.6±7.9	60.6±7.5^‡^	61.1±8.0^‡^	60.5±7.7^‡^	<0.0001
						
Sex	Male	2321 (59)	153 (48)	119 (45)	185 (74)	<0.0001
	Female	1640 (41)	168 (52)	147 (55)	65 (26)	
						
Antihypertensive use	No	2416 (61)	196 (61)	144 (54)	164 (66)	0.059
	Yes	1545 (39)	125 (39)	122 (46)	86 (34)	
						
Diabetes	Yes	67 (2)	13 (4)	9 (3)	11 (4)	0.0005
	No	3894 (98)	308 (96)	257 (97)	239 (96)	
						
Cardiovascular disease	Yes	495 (13)	32 (10)	30 (11)	29 (12)	0.55
	No	3466 (88)	289 (90)	236 (89)	221 (88)	
						
Heavy alcohol consumption	Never	228 (6)	23 (7)	24 (9)	3 (1)	<0.0001
(⩾6 drinks per occasion)	⩽1 per month	1037 (26)	58 (18)	91 (35)	26 (11)	
	Weekly, daily or almost daily	2669 (68)	237 (75)	146 (56)	215 (88)	
						
Smoking	Non-smoker	2024 (51)	172 (54)	76 (29)	170 (69)	<0.0001
	Ex-smoker	1728 (44)	106 (33)	140 (53)	69 (28)	
	Current smoker	201 (5)	42 (13)	48 (18)	9 (4)	
						
Body mass index (kg m^−2^)		27.9±4.5	33.8±6.1^‡^	31.8±6.6^‡^	27.0±4.1^†^	<0.0001
						
Pulse rate (beats per minute)		62.9±9.8	64.6±10.2^†^	63.9±11.0	68.0±11.1^‡^	<0.0001

^†^*P*<0.01, ^‡^*P*<0.001 compared with European.

a Values are sample size (column %) or mean±s.d.

**Table 2:** Differences in arterial waveform parameters between European and non-European groups^a^

*Parameter*	*Mean (s.e.)*	*Difference compared with European (95% confidence interval)*			P*-value*
	*European (*n=*3961)*	*Pacific (*n=*321)*	*Maori (*n=*266)*	*South Asian (*n=*250)*	
*Unadjusted for brachial BP*					
Brachial SBP (mm Hg)	137.4 (0.4)	**5.4 (3.3, 7.6)**	**3.8 (1.5, 6.1)**	0.1 (−2.3, 2.5)	***P*****<0.0001**
Brachial DBP (mm Hg)	76.7 (0.2)	**2.5 (1.4, 3.7)**	**2.6 (1.4, 3.9)**	−1.1 (−2.4, 0.2)	***P*****<0.0001**
Aortic SBP (mm Hg)	129.1 (0.4)	**5.3 (3.2, 7.3)**	**3.9 (1.6, 6.1)**	0.5 (−1.8, 2.9)	***P*****<0.0001**
Augmentation index	29.1 (0.2)	0.1 (−1.0, 1.3)	**1.8 (0.5, 3.0)**	**4.0 (2.7, 5.3)**	***P*****<0.0001**
Backward pressure amplitude (mm Hg)	20.8 (0.1)	**1.0 (0.3, 1.6)**	**0.8 (0.0, 1.5)**	**0.9 (0.1, 1.6)**	***P*****=0.003**
Peak reservoir pressure (mm Hg)	119.9 (0.4)	**4.4 (2.5, 6.3)**	**3.5 (1.5, 5.6)**	0.3 (−1.9, 2.4)	***P*****<0.0001**
log_e_(excess pressure integral (mm Hg.s))	1.29 (0.01)	**0.13 (0.09, 0.18)**	**0.07 (0.02, 0.11)**	**0.05 (0.00, 0.10)**	***P*****<0.0001**
Pulse wave velocity (m s^−1^)	9.47 (0.02)	**0.12 (0.03, 0.21)**	0.07 (−0.02, 0.17)	0.00 (−0.10, 0.11)	**0.042**
					
*Adjusted for brachial SBP*					
Aortic SBP (mm Hg)	130.7 (0.1)	0.1 (−0.4, 0.6)	0.3 (−0.2, 0.8)	0.4 (−0.1, 1.0)	0.32
Augmentation index	29.5 (0.2)	−**1.2 (**−**2.3, -0.2)**	0.8 (−0.3, 2.0)	**3.9 (2.8, 5.1)**	**<0.0001**
Backward pressure amplitude (mm Hg)	21.2 (0.09)	−0.3 (−0.8, 0.1)	−0.1 (−0.6, 0.4)	**0.8 (0.3, 1.3)**	**0.004**
Peak reservoir pressure (mm Hg)	121.3 (0.2)	0.0 (−0.8, 0.8)	0.5 (−0.4, 1.4)	0.2 (−0.7, 1.0)	0.76
log_e_(excess pressure integral (mm Hg.s))	1.30 (0.01)	**0.08 (0.04, 0.11)**	0.03 (−0.01, 0.07)	**0.05 (0.01, 0.09)**	**<0.0001**
Pulse wave velocity (m s^−1^)	9.53 (0.01)	−**0.09 (**−**0.13,** −**0.05)**	−**0.07 (**−**0.11,** −**0.03)**	0.00 (−0.04, 0.04)	**<0.0001**
					
*Adjusted for brachial DBP*					
Aortic SBP (mm Hg)	131.0 (0.3)	**2.0 (0.6, 3.4)**	0.5 (−1.0, 2.1)	**2.0 (0.4, 3.6)**	**0.007**
Augmentation index	29.6 (0.2)	−0.8 (−1.8, 0.3)	0.8 (−0.4, 2.0)	**4.7 (3.5, 5.9)**	**<0.0001**
Backward pressure amplitude (mm Hg)	21.0 (0.1)	0.6 (−0.0, 1.3)	0.4 (−0.3, 1.1)	**1.0 (0.3, 1.7)**	**0.018**
Peak reservoir pressure (mm Hg)	121.7 (0.2)	1.2 (-0.0, 2.4)	0.2 (−1.1, 1.5)	**1.7 (0.3, 3.0)**	**0.034**
log_e_(excess pressure integral (mm Hg.s))	1.29 (0.01)	**0.13 (0.09, 0.17)**	**0.07 (0.02, 0.11)**	**0.05 (0.00, 0.10)**	**<0.0001**
Pulse wave velocity (m s^−1^)	9.52 (0.02)	0.02 (−0.06, 0.09)	−0.03 (−0.12, 0.05)	0.05 (−0.04, 0.14)	0.50

Abbreviations: BP=blood pressure; DBP=diastolic BP; SBP=systolic BP.

a All models were adjusted for age, sex, antihypertensive use, diabetes and cardiovascular disease, plus pulse rate for augmentation index. Some models were further adjusted for brachial SBP or brachial DBP, as indicated in italics above. *P*-values in table are for main effects. 95% confidence intervals that do not encompass 0 and significant main effects (*P*<0.05) for waveform variables are in bold.

**Table 3:** Relationships between modifiable lifestyle factors and arterial waveform parameters^a^

*Modifiable factor and level*	*Arterial waveform parameter*						
	*Brachial systolic blood pressure (mm Hg)*	*Aortic systolic blood pressure (mm Hg)*	*Augmentation index*	*Backward pressure amplitude (mm Hg)*	*Peak reservoir pressure (mm Hg)*	*log*_*e*_*(excess pressure integral (mm Hg s))*	*Pulse wave velocity (m s^−1^)*
*Heavy alcohol consumption*							
Never^b^	139.3 (0.6)	131.0 (0.6)	30.4 (0.3)	21.34 (0.18)	121.6 (0.5)	1.34	9.50 (0.02)
⩽1 per month^c^	**1.7 (0.4, 2.9)**	**1.6 (0.4, 2.9)**	0.5 (−0.2, 1.2)	**0.41 (0.0, 0.82)**	**1.5 (0.4, 2.7)**	(0.01) 0.02 (−0.01, 0.04)	**0.06 (0.01, 0.12)**
Weekly, daily or almost daily^c^	**4.2 (1.9, 6.5)**	**3.9 (1.6, 6.1)**	1.0 (−0.3, 2.2)	0.34 (−0.39, 1.08)	**3.2 (1.1, 5.2)**	0.03 (−0.01, 0.08)	**0.14 (0.04, 0.23)**
	***P*****=0.0003**	***P*****=0.0005**	*P*=0.19	*P*=0.13	***P*****=0.0013**	*P*=0.19	***P*****=0.0046**
							
*Smoking*							
Non-smoker^b^	139.1 (0.6)	130.8 (0.6)	29.9 (0.3)	21.32 (0.20)	121.6 (0.6)	1.34 (0.01)	9.50 (0.03)
Ex-smoker^c^	**1.2 (0.1,2.3)**	**1.3 (0.2,2.3)**	**0.6 (0.0, 1.2)**	0.23 (−0.11,	0.8 (−0.1,1.8)	0.02 (−0.00, 0.04)	**0.05 (0.01,0.10)**
Current smoker^c^	1.3 (−0.9, 3.5)	1.1 (−1.0, 3.3)	**3.7 (2.5, 4.9)**	0.58) 0.10 (−0.61, 0.81)	0.2 (−1.8, 2.2)	0.04 (−0.01, 0.08)	−0.01 (−0.10, 0.08)
	*P*=0.07	*P*=0.05	***P*****<0.0001**	*P*=0.42	*P*=0.23	*P*=0.08	*P*=0.070
							
Body mass index (kg m^−2^)	**0.57 (0.46, 0.68)**	**0.51 (0.40, 0.62)**	**−0.2 (−0.3, −0.2)**	0.03 (−0.01, 0.06)	**0.5 (0.4, 0.6)**	**0.005 (0.002, 0.007)**	**0.02 (0.01, 0.02)**
	***P*****<0.0001**	***P*****<0.0001**	***P*****<0.0001**	*P*=0.17	***P*****<0.0001**	***P*****<0.0001**	***P*****<0.0001**

a Adjusted for age, sex, ethnicity, antihypertensive use, diabetes and cardiovascular disease, plus pulse rate for augmentation index; *P*-values listed are for main effects.

b Reference group—values are adjusted means (95% confidence intervals).

c Values are differences (increments) in adjusted means (95% confidence intervals); *P<0.05, ^†^*P*<0.01, ^‡^*P*<0.001 compared with reference group (for categorical variables) or for the slope (for BMI); 95% confidence intervals that do not encompass 0 and significant main effects (*P*<0.05) for waveform variables are in bold.
