# Erratum: Arterial waveform parameters in a large, population-based sample of adults: relationships with ethnicity and lifestyle factors

**DOI:** 10.1038/jhh.2017.60

**Published:** 2017-08-31

**Authors:** J D Sluyter, A D Hughes, SAMcG Thom, A Lowe, C A Camargo, B Hametner, S Wassertheurer, K H Parker, R K R Scragg

**Correction to:** *Journal of Human Hypertension* (2017) **31**, 305–312; doi:10.1038/jhh.2016.78; published online 22 December 2016

The text of the licence type for this paper was incorrect on the full text version. The following is the correct text:

The publishers wish to apologise for their error.

